# Behind the Screen: An Exploratory Study of Factors Influencing Breast Screening Uptake in Lancashire (UK)

**DOI:** 10.1111/hex.70183

**Published:** 2025-02-17

**Authors:** Yik Nok Bryan Lee, Alexander Montasem, Lauren Haworth, Jonathan Sinclair, Kim McGuire, Ambreen Chohan

**Affiliations:** ^1^ Allied Health Research Unit University of Central Lancashire Preston UK; ^2^ School of Medicine University of Central Lancashire Preston UK; ^3^ School of Dentistry The University of Liverpool Liverpool UK; ^4^ Centre for Applied Sport, Physical Activity and Performance University of Central Lancashire Preston UK

**Keywords:** barriers, breast, breast cancer, public health, screening, socioecological

## Abstract

**Background:**

Breast screening uptake has improved nationally to 62% in the United Kingdom, though regionally, engagement challenges remain in northern regions such as Lancashire (54%–59%). It is important, therefore, to understand the barriers women face to enable appropriate person‐ and community‐centred engagement in health screening behaviours.

**Objectives:**

This study aimed to be the first mixed‐method questionnaire exploration of women in Lancashire (the United Kingdom) to explore attitudes, behaviour, awareness, barriers and facilitators to breast screening.

**Design:**

Cross‐sectional cohort study.

**Method:**

The Breast Cancer Fear Scale, modified Mammography Self‐Efficacy Scale and the General Practice Physical Activity Questionnaire were included in the questionnaire alongside open‐ended elements on breast screening behaviour and awareness. Registered female participants (*n* = 50) were provided with digital assistance or language interpretation where requested.

**Results:**

Ethnicity, faith and location all affected perceived levels of breast screening awareness, yet only age and faith influenced understanding of the process. Irrespective of protected characteristics, fear of breast cancer did not significantly vary between women. Racially minoritised women were less comfortable in removing clothing during screening. Participants reported barriers related to health awareness, patient experience, screening age and access to healthcare. Facilitators to address barriers were identified using a socioecological framework to identify key areas of development needed at an individual, interpersonal, organisational, community and public policy level.

**Conclusion:**

Protected characteristics and geographical location significantly influence breast screening behaviour. Targeted person‐centred health awareness, cultural competency and inclusive practice are needed to promote awareness, remove taboos and open up dialogue and acceptance of breast cancer screening in communities. The use of the socioecological model highlighted that the responsibility to reduce barriers to breast screening in Lancashire is collective from an individual to public policy level. Further patient–public involvement would ensure adequate demographic representation and effectively investigate differences between ethnic subgroups.

**Patient or Public Contribution:**

This article captures the viewpoints of individuals with and without experience of the breast screening process in the United Kingdom. A small group of individuals from white and racially minoritised backgrounds were involved in the design of the study to ensure the suitability and acceptability of the tool.

## 1 Introduction

Breast cancer surpassed lung cancer as the leading cause of global cancer incidence in 2020, with an estimated 2.3 million new cases, representing a quarter of all cancers in females [1]. If current trends remain unchanged, the burden of breast cancer is set to grow to over 3 million new cases and 1 million deaths per year by 2040 as a result of population growth and ageing alone [2].

In the United Kingdom, women between the ages of 50 and 71 are invited to breast screening from the National Health Service (NHS) every 3 years, through the NHS Breast Screening Programme (NHSBSP) [3, 4]. Whilst screening has increased survival rates from 40% in the 1970s to 76% between 2013 and 2017 [5], nationally only 61.8% of age‐eligible women attended breast screening in 2020–2021. This was a 7.3% decrease compared to the previous year, due to the likely impacts of Covid‐19 [6]. The first women's health strategy for England has recently been published, to address gender‐related health inequalities [7]. Its implementation strategy for the £10‐million investment involves increasing the number of mobile screening units to overcome low screening uptake, as well as scheduling immediate breast reconstruction for women post‐mastectomy [7]. Ultimately though, the effectiveness of this new strategy and technological advancement to improve breast health is reliant upon screening uptake. At an organisational level, difficulty in booking screening appointments may discourage attendance [8]. Reasons behind this may be multifaceted, relating to logistics, site location and access, up‐to‐date contact information, effective booking systems and training [9, 10, 11, 12]. Therefore, simply removing organisational barriers in the NHS and improving access/location may only partially solve uptake rates. To ensure higher screening uptake, increased efforts must be made to understand and identify all relevant barriers to screening, to develop person‐ and community‐centred solutions.

It has been suggested that women with greater breast health awareness are more likely to attend breast and cervical screening [13]. Socioecological barriers associated with age, knowledge, awareness, cultural beliefs, language, socioeconomic status and disability have previously been identified [11, 14, 15, 16, 17, 18, 19, 20, 21, 22, 23, 24, 25, 26, 27, 28]. The term ‘cancer’ and its perception have often been subject to stigma in ethnica minoritised communities, with health beliefs and cognitive representation of disease often a confounding factor. Some women, for example, believe that cancer could be spread through close human contact and that simply mentioning the word ‘cancer’ could put the person at risk of developing it [29, 30]. The stigma of breast cancer in the family has also been negatively associated with marriage, with women reporting marital breakdown due to cancer and others reporting an impact on the extended family by negatively influencing the marriage prospects of their children [30, 31]. Other reported cultural barriers limiting cancer and breast health knowledge include women being prevented from attending events related to breast cancer in a male‐dominated family unit [33]. Whilst the incidence rate for breast cancer amongst racially minoritised groups has been shown to be lower than white individuals, they have often been diagnosed at a later stage, with poorer prognosis [29, 33]. Many have suggested that low or delayed uptake in breast screening may contribute to delayed diagnosis [24, 34, 35, 36, 37]. In previous studies, black women identified breast cancer as a disease associated with non‐minoritised groups, suggesting a lower perceived risk of developing the disease [38, 39, 40].

Individuals with spiritual or religious beliefs have also been shown to relate cancer to the stigma of punishment for any previous wrongdoing [30, 40]. The stigma of this incurable and ‘unspeakable’ disease may result in a reduction of health‐seeking behaviour by many women of breast screening age, resulting in later diagnosis, and reduced chance of survival, or practice of alternative medicine to delay acceptance of symptoms [41, 42]. Faith and emotional barriers such as embarrassment in removing clothing for screening, screening practitioner gender barriers, and pain and discomfort experienced during the screening procedure previously have all impacted breast screening attendance rates [14, 25, 43, 44, 45, 46, 47, 48, 49], whilst moderate levels of worry have been shown to act as a motivator to attend [50].

Additional drivers of screening behaviours are related to the wider determinants of health. For example, higher education levels and income have been shown to have a positive association with breast screening attendance [25], whilst screening site location, accessibility and cost associated with travel have had a negative influence on screening uptake, particularly when salary or time loss could not be compensated [8, 19, 51, 52]. Language barriers impact health‐seeking behaviour amongst ethnically minoritised groups [39, 53, 54], thus adversely impacting the relationship between healthcare professionals (HCPs) and patients. The associated negative attitudes from HCPs may also decrease the likelihood of screening reattendance [46, 55].

Understanding regional and local barriers to breast screening behaviours is vital for reducing health inequalities and increasing uptake. The 2021 census showed that 11.1% of the population in Lancashire comprised ethnically minoritised groups [56]. Breast screening attendance in Lancashire is lower (53.9%–59.4% of eligible women) than the national screening rate (61.8%) [3, 6]. Studies performed specifically in Lancashire to investigate the barriers leading to low screening uptake are limited, although language, cultural beliefs and breast screening knowledge have been identified as major barriers to screening uptake for British‐Pakistani women from East Lancashire [57]. With diversity in the region increasing, it is important to understand the true needs of the community accessing breast screening services to help reduce cancer‐related health inequities. This study is the first to use a mixed‐methods questionnaire with a diverse range of women in Lancashire (the United Kingdom), to understand the behaviour, awareness, barriers and facilitators to breast screening to help guide trust‐based health promotion initiatives.

## 2 Methods

### 2.1 Study Design and Sample Size

The study design was an exploratory cross‐sectional cohort study incorporating a mixed‐methods questionnaire. Consistent with published rules of thumb for pilot studies, a minimum of 20 healthy participants were recruited per area [58, 59].

### 2.2 Participants

Volunteers were recruited independently using opportunity sampling at three health promotion events in Lancashire, run in partnership with two Lancashire‐based NHS trusts between March and June 2022. Breast cancer awareness interventions have been found to increase the uptake of breast self‐examination behaviour and increase the likelihood of breast screening attendance [60]. It has also been postulated that adolescence is a critical period where lifelong behaviours form and are maintained into adulthood [61]. Therefore, this study's inclusion criteria stipulated that only volunteers aged over 18 years, registered as female on their medical records [62], were eligible to participate in this study, in line with guidance from the NHSBSP [63]. The authors included ages below and above the standard screening age range as it was important to understand that the likelihood of self‐attendance or influencing others may be based on lived experience and opinions. University ethics approval was obtained (HEALTH0293). All data collection conformed to the Declaration of Helsinki [64] and General Data Protection Regulations, and informed written anonymised consent was obtained before participation, with individuals able to withdraw only up to the point of submission.

### 2.3 Data Collection

Generated using Microsoft Forms, this questionnaire was designed with input from Equality, Diversity and Inclusion teams at the University and local NHS trusts in the region, patient experience teams (NHS trusts), clinicians and a Patient and Public Involvement (PPI) group. The questionnaire was structured into two main domains with elements focusing on: (1) the individual, including physical activity behaviour, individual characteristics (age, main language, faith, disability/learning difficulty, ethnicity) and demographics (geographical region by postcode), and (2) breast screening behaviour/intention and awareness. Psychometrically validated self‐reported questionnaires including the ‘Breast Cancer Fear Scale’ (BCFS) [65], modified ‘Mammography Self‐Efficacy Scale’ (MSES) [66] and the ‘General Practice Physical Activity Questionnaire’ (GPPAQ) [67] were used as outcome measures to assess the need for service improvement within the region. Permission was sought from the authors for the use and adaptation of BCFS and MSES [65]. Open‐response items were used to explore potential factors influencing breast screening uptake. Where required, the questionnaire was verbally translated in languages appropriate to the demographics at the different events (Hindi, Urdu, Punjabi, Bengali, Pushto), to enable verbatim collection of data from diverse communities. All translators were briefed accordingly. Individuals were able to use their own mobile devices to access the questionnaire via a QR code or were provided with a tablet to complete the questionnaire. Assistance from researchers was provided to complete the questionnaires, where participants requested. As multiple questions required routing or were optional, a response was not required by everyone for every question. However, due to the anonymity of the study, participants were encouraged to participate and feed back openly.

### 2.4 Data Analysis

The validated components of the questionnaire were analysed according to previously used methods [65, 66, 67].

#### 2.4.1 Champion BCFS

Champion BCFS provides a framework for subjective emotional responses. The *categorical* scale captures an individual's fear of cancer and has been shown to predict breast screening behaviour [65]. Each question in this 8‐item tool was rated using a Likert scale from (1) ‘I strongly disagree’ to (5) ‘I strongly agree’, resulting in a possible score of between 8 and 40. Levels of fear were categorised as low‐level (8–15 points), mid‐level fear (16–23 points) and high‐level fear (24–40 points).

#### 2.4.2 Modified MSES

This tool was modified by removing the question related to the cost of mammography due to the NHSBSP being free at the point of access. This 9‐item tool used a *numerical* scale from 1 to 5, with a maximum possible score of 45 possible to assess an individual's likelihood of attending mammography screening. A high score indicated an increased likelihood of breast screening attendance [66].

#### 2.4.3 GPPAQ

GPPAQ is a screening tool used in general practice settings to assess an individual's physical activity level. Four‐level Physical Activity Indexes (PAI) were calculated based on three questions from the questionnaire. Individuals were then *categorised* into four groups, varying from inactive to active. This tool has previously been used by GPs to provide specific advice and recommendations for physical health improvement [67].

#### 2.4.4 Breast Screening Behaviour/Intention and Awareness

Closed questions in this section related to participants' breast screening intention and awareness. (1) Have you booked and attended or are you planning to attend a breast screening appointment? (2) Do you feel comfortable removing your clothing for breast screening? (3) How aware are you of the NHSBSP? (4) Do you understand what will happen when you come for a breast screening appointment? (5) How often, if ever, do you carry out a self‐examination of your breasts for any change? (6) Do you have any worries about breast screening or does anything put you off going for a breast screening appointment?

### 2.5 Barriers and Facilitators to Breast Screening

Two open‐response questions were used to establish barriers and facilitators to accessing breast screening. Utilising open‐response questions elicits the individual's understanding of various factors. Due to the exploratory nature of this study, open‐response items through the questionnaire afforded thematic analysis. An inductive/deductive approach was adopted, allowing the data to generate the themes and then framing them using the socioecological systems theory to highlight structural barriers and key stakeholders influencing the breast screening journey [68, 69]. Thematic analysis is a widely used tool in qualitative analysis, despite some ambiguities in the theoretical, epistemological or other approaches accompanying its use [70, 71, 72]. Hence, it is crucial to overt the reasons for using thematic analysis. A socioecological systems theory was applied to illustrate the dynamic interplay between micro‐ and macro‐level factors contributing to inequities in breast screening uptake and to discuss implications for the reduction of inadequate access [73]. The approaches above focussed on the key elements of the *individual*, *interpersonal* relationships or interaction, *organisational factors*, or *public policy*. Our work sought to investigate the perceived mechanisms contributing to access and understanding of breast screening.

### 2.6 Statistical Analysis

All data were exported to Microsoft Excel for processing. SPSS Statistics 28 (IBM, New York, the United States) was used to analyse the quantitative components of the data. Descriptive statistics of means and standard deviations were reported for continuous data and number of participants (*n*) and % for categorical data. For continuous numerical data, one‐way univariate ANOVA were adopted with significant main effects further explored using pairwise comparisons with LSD confidence interval adjustments. Relationships between individual characteristics (i.e., age, ethnicity, faith and area) were explored for each of the validated questionnaires (i.e., GPPAQ, BCFS and MSES). Two‐way Pearson *χ* ^2^ tests of independence were used to undertake bivariate cross‐tabulation comparisons, specifically to test differences in responses to each categorical question between demographic/individual characteristic variables. Though language was recorded in the questionnaire to maintain anonymity, this was excluded in the secondary analysis of the data due to the low response rate (*n* < 5) in each group. The probability value threshold for statistical significance for both continuous and categorical data analyses was accepted at the *p* ≤ 0.05 level.

## 3 Results

### 3.1 Individual Characteristics

Fifty participants were included in this study and completed the questionnaire either independently (*n* = 33), received assistance from researchers on request (*n* = 10) or received assistance due to language interpretation requirements identified on recruitment (*n* = 7). As all data was captured verbatim and all translators were briefed and trained, it was not envisaged that this impacted data collection. Participant characteristics are reported in Table 1.

**Table 1:** Characteristics of participants.

Characteristic	Groups	*n* (%)
Age	< 39	13 (26%)
	40–50	17 (34%)
	51–60*	6 (12%)
	61–70*	7 (14%)
	> 71	7 (14%)
Main language	Bengali	1 (2%)
	English	42 (84%)
	Pashto	2 (4%)
	Punjabi	2 (4%)
	Turkish	1 (2%)
	Urdu	2 (4%)
Faith	Christian	14 (28%)
	Muslim	24 (48%)
	No faith	10 (20%)
	Prefer not to say	2 (4%)
Disability/learning difficulty	No	37 (74%)
	Prefer not to say	1 (2%)
	Registered deaf/hearing impairment	1 (2%)
	Mental health condition	4 (8%)
	Disability affecting mobility/physical impairment	4 (8%)
	Other (e.g., neurodiverse, cognitive)	3 (6%)
Ethnicity	Asian/Asian British	*n* = 23 (46%)
	White	*n* = 27 (54%)
Area	East Lancashire	*n* = 26 (52%)
	South Lancashire	*n* = 24 (48%)

* The age ranges within the current screening age range.

### 3.2 GPPAQ

Most participants (42%) were unemployed (e.g., retired, retired for health reasons, unemployed and full‐time carer). Working women were divided across sectors, with 20% involved in a sedentary occupation (e.g., office work), 14% in a physical occupation (e.g., nurse, gardener, electrician, scaffolder and construction worker) and the majority (24%) with an occupation that involved standing or walking (e.g., shop assistant, hairdresser, security guard, child‐minder, etc.).

Women categorised as unemployed were deemed to have a lower PAI in comparison to other occupation groups, suggesting less engagement with physical activity. GPPAQ scores were significantly influenced only by age, with younger women being more active compared to older individuals (*p* = 0.017). However, GPPAQ appears to have a discriminatory effect on people in retirement as this group of individuals was identified as inactive, without accounting for time spent walking, gardening or doing housework each week. To consider the proportion of participants that were truly inactive, guidance from the International Physical Activity Questionnaire was incorporated to account for walking. Analysis showed only 22% were truly inactive when compared to 42% when using GPPAQ alone.

### 3.3 MSES

MSES scores are reported in Table 2, with further statistical analysis in Table 3. MSES scores were not influenced by age (*p* = 0.065). Women from South Lancashire scored significantly higher in the MSES compared to women from East Lancashire (*p* < 0.001), indicating a greater likelihood of screening attendance. White women felt significantly more confident in attending mammography screening compared to those women of Asian/Asian British heritage (*p* = 0.001).

**Table 2:** Mean (standard deviation) results for MSES and categorical data for GPPAQ and BCFS [*n* (%)].

Characteristic	Category	MSES	GPPAQ *n* (%)				BCFS *n* (%)		
		Mean (SD)	Inactive	Mod. inactive	Mod. active	Active	Low fear	Moderate fear	High fear
Faith	Christian	40.4 (4.9)	11 (22%)	1 (2%)	0 (0%)	2 (4%)	6 (12%)	4 (8%)	4 (8%)
	Muslim	30.5 (11.4)	10 (20%)	6 (12%)	2 (4%)	6 (12%)	8 (16%)	7 (14%)	9 (18%)
	No faith	40.5 (4.7)	6 (12%)	0 (0%)	2 (4%)	2 (4%)	7 (14%)	1 (2%)	2 (4%)
	Prefer not to say	24.0 (9.9)	0 (0%)	0 (0%)	0 (0%)	2 (4%)	0 (0%)	1 (2%)	1 (2%)
Age	≤ 39	35.8 (8.9)	4 (8%)	1 (2%)	1 (2%)	7 (14%)	6 (12%)	4 (8%)	3 (6%)
	40–50	30.1 (12.1)	6 (12%)	6 (12%)	2 (4%)	3 (6%)	5 (10%)	6 (12%)	6 (12%)
	51–60*	34.7 (7.3)	4 (8%)	0 (0%)	1 (2%)	1 (2%)	2 (4%)	1 (2%)	3 (6%)
	61–70*	38.4 (9.1)	6 (12%)	0 (0%)	0 (0%)	1 (2%)	3 (6%)	0 (0%)	4 (8%)
	≥ 71	42.3 (3.5)	7 (14%)	0 (0%)	0 (0%)	0 (0%)	5 (10%)	2 (4%)	0 (0%)
Ethnicity	White	39.1 (6.5)	17 (34%)	2 (4%)	2 (4%)	6 (12%)	13 (26%)	6 (12%)	8 (16%)
	Asian/Asian British	30.2 (11.6)	10 (20%)	5 (10%)	2 (4%)	6 (12%)	8 (16%)	7 (14%)	8 (16%)
Area	South Lancashire	40.5 (5.1)	16 (32%)	2 (4%)	2 (4%)	4 (8%)	10 (20%)	6 (12%)	8 (16%)
	East Lancashire	30.0 (11.0)	11 (22%)	5 (10%)	2 (4%)	8 (16%)	11 (22%)	7 (14%)	8 (16%)

Abbreviation: Mod. = moderately.

* The age ranges within the current screening age range.

**Table 3:** Main statistical outcomes for all quantitative data relative to participant demographics.

Characteristic	MSES	GPPAQ	BCFS	Booked/planning to attend NHSBSP	Clothing removed for breast screening	Screening awareness	Understanding of breast screening	Regular self‐examination in line with national advice	Breast screening concerns
Faith^A^	0.001*	0.064	0.466	0.077	0.070	0.007*	0.043*	0.753	0.459
Age	0.065	0.017*	0.296	< 0.001*	0.666	0.558	0.027*	0.795	0.821
Ethnicity	0.001*	0.424	0.621	0.087	0.044*	0.003*	0.089	0.384	0.099
Area	< 0.001*	0.325	0.978	0.055	0.114	0.013*	0.089	0.253	0.067

* Significance was set to ≤ 0.05.

^A^ ‘Prefer not to say’ group in faith was not relevant to the comparison, so it was not reported.

### 3.4 BCFS

Forty‐two percent (*n* = 21) of women reported a low fear of breast cancer, whilst 26% (*n* = 13) experienced moderate fear and 32% (*n* = 16) reported a high fear. Comparisons were subsequently analysed between the three validated questionnaires and other variables to test for association and significance. Individual characteristics included in this study did not influence BCFS scores (*p* > 0.29; Table 3).

### 3.5 Breast Screening Behaviour/Intention and Awareness

When asked about their level of awareness about the NHSBSP, significantly more white women perceived themselves to be very aware (Tables 3 and 4; *p* = 0.003). Christian women were generally very aware about NHSBSP compared to women of Muslim faith and those with no reported faith (*p* = 0.007). NHSBSP awareness in women from South Lancashire was greater than women from East Lancashire (*p* = 0.013).

**Table 4:** Breast screening behaviour, awareness and concerns.

Characteristic		Booked/planning to attend NHSBSP	Clothing removed for breast screening			Breast screening awareness		Understanding of breast screening			Regular Self‐examination in line with national advice		Breast cancer screening concerns	
		Yes	Yes	No	Not sure	A little to no awareness	Very aware	Yes	No	Not sure	Yes	No	Yes	No
Faith^A^	Christian	9	10	4	0	2	12	13	1	0	13	1	2	12
	Muslim	4	11	12	1	17	7	11	7	6	15	7	8	16
	No faith	5	8	0	2	3	7	7	0	3	9	1	2	8
Age	≤ 39	1	6	5	2	8	5	5	3	5	9	3	2	11
	40–50	1	9	7	1	9	8	8	6	3	13	3	5	12
	51–60*	4	4	2	0	4	2	6	0	0	5	1	2	4
	61–70*	6	5	2	0	1	6	6	0	1	6	1	2	5
	≥ 71	6	6	1	0	1	6	7	0	0	6	1	1	6
Ethnicity	White	14	20	5	2	7	20	21	3	3	24	3	4	23
	Asian/Asian British	4	10	12	1	16	7	11	6	6	15	6	8	15
Area	South Lancashire	13	18	5	1	6	18	19	3	2	22	2	3	21
	East Lancashire	5	12	12	2	17	9	13	6	7	17	7	9	17

^A^ ‘Prefer not to say’ group was not included in Faith table as faith was not declared.

* The age ranges within the current screening age range.

When asked whether they understood the breast screening process, age and faith were both influencing factors (*p* < 0.05; Tables 3 and 4). Significantly more older women (aged ≥ 50) reported an understanding of the breast screening process compared to younger individuals. A greater proportion of women of Christian faith (93%) and no faith (70%) reported understanding the screening process compared to women of Muslim faith (46%).

Thirty‐six percent of women responded that they had booked or were planning to attend their screening appointment. Ethnicity was found to influence women's decisions on whether they felt comfortable removing clothing for screening (*p* = 0.044). Fifty‐seven percent of Asian/Asian British women did not feel comfortable or were unsure about removing clothing compared to 26% of white women.

### 3.6 Barriers and Facilitators to Breast Screening

**Figure 1:**
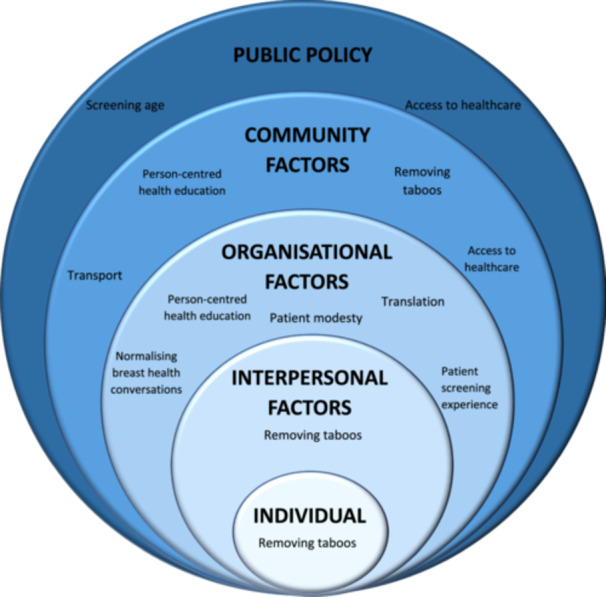
Socioecological model of factors influencing breast screening behaviour adapted from [68].

Responses to open‐response items (56% response rate) explored perspectives on facilitators to reduce barriers to breast screening. Responses were coded manually, and core themes were established: Patient‐centred health awareness, patient experience, screening age and access to healthcare. The socioecological model was used to classify the themes into five key influencing factors: Individual, Intrapersonal, Organisational, Community and Public policy factors [68] (Figure 1).

#### 3.6.1 Patient‐Centred Health Awareness

Multiple individuals suggested the need to improve awareness, which included *organisation* lead, *community* focussed initiatives, advertising and targeted awareness days.

‘Having a check your breast day’ [P6]

‘More advertising through GP surgery’ [P10]

‘Encourage more people….’ [P50]

Others were more focussed on the need for targeted awareness raising in their communities.

‘More awareness around different areas and Muslim centres such as colleges or Sahara [voluntary women's organisation] or Madrassa [religious schools]’ [P47]

‘Need more awareness in my Asian community.’ [P18]

‘Target small communities, raise awareness, embed better with the communities.’ [P48]

Some individuals suggested that the information was not provided in a suitable format for them and suggested that translated information should be more widely offered, for example, in the Urdu language. Women also suggested that there was a need to remove taboos around breast health at an *individual, interpersonal and community* level.

‘…encourage boobs as a normal, not a thing to be embarrassed about. Breasts are usually sexualised and that makes some women uncomfortable and embarrassed.’ [P28]

#### 3.6.2 Patient Experience

Some women highlighted *organisational* challenges around patient experience indicating pain of the screening process as a barrier.

‘Quite painful palpation, it feels like it will damage cells.’ [P47]

‘Pain. Depends who does it. Puts women off when it is painful’ [P50]

Others made suggestions to try and improve the experience, suggesting a need for reassurance and a need to develop procedures that were less painful or more comfortable.

‘Try and make more comfortable during the screening appointments’ [P16].

Other suggestions were made around the temperature and experience of the screening vehicles and patient modesty

‘…heating the vehicles’ [P48]

‘The whole process should be possible wearing a patient gown for it. It isn't fair to assume women are okay with the process.’ [P37]

#### 3.6.3 Screening Age

With the national guidance on screening age being part of national *public policy*, women also raised concern around the screening age with some suggesting that it should be lowered and others suggesting it should be extended past the age of 70 as age was not a barrier.

‘Younger offering. Recent friends diagnosed at 37 and 26 after struggling with pain…’ [P36]

‘Screening up to 80 as there is no age barrier’ [P47]

‘…increase the age range’ [P48]

#### 3.6.4 Access to Healthcare

Two women suggested that there were challenges around accessing healthcare, directly linking to *public policy* challenges.

‘…. more GP's.’ [P50]

‘NHS is horrible for appointments too far in the future. Cry to be referred to secondary care.’ [P49]

A suggestion was also made to improve access through the provision of transport at a *community* level.

‘Car or transport to get there’. [P17]

## 4 Discussion

This study aimed to be the first mixed‐methods questionnaire exploration of attitudes, behaviours/intentions, awareness, barriers and facilitators associated with breast screening amongst a diverse range of women in Lancashire (the United Kingdom). To the authors' knowledge, it was the first study to combine a battery of validated questionnaires (BCFS, MSES and GPPAQ) as measures of breast screening knowledge and open‐response questions to identify barriers and facilitators of breast screening attendance in Lancashire (the United Kingdom).

Individual characteristics, including age, ethnicity, faith and geographical location, were all found to significantly influence breast screening behaviours (MSES) and physical activity levels (GPPAQ). Though the results for MSES suggested that there was a significantly higher likelihood of screening attendance in women from South Lancashire compared to East Lancashire, it is important to consider that this is not yet a representative sample of all women in these areas from a demographic perspective.

Due to potential bias in reliability and accuracy in the use of GPPAQ, modifications should be made around this element in future studies. This is primarily due to the GPPAQ tool not clearly reflecting the experience of unemployed or retired individuals. Fear of breast cancer (BCFS) was not influenced by individual characteristics [29].

### 4.1 Breast Screening Behaviour and Awareness

Current breast screening attendance rates in Lancashire are at approximately 53.9%–59.4% [3, 6] amongst age‐eligible women, and the findings from this study demonstrate a similar likelihood of attendance rates at 62%. Such low screening uptake has the potential to exacerbate cancer‐related health inequities amongst women.

Women aged ≥ 50 reported a better understanding of the breast screening process compared to younger individuals, although this may be attributed to hands‐on experience versus no experience. Most white women perceived themselves to be very aware of the NHSBSP, whilst most Asian/Asian British women perceived themselves to have little to no awareness of the NHSBSP and process. Whilst the reasons for awareness levels were not explored in the present study, there has been a growing body of evidence associating levels of cancer awareness with delayed presentation, poorer survival and late diagnosis [34, 74, 75, 76]. This highlights the need to focus further health promotion activities towards women earlier than the screening age and, in particular, targeting health awareness strategies towards racially minoritised groups.

Faith has been shown in this study to influence screening awareness and behaviour, with greater proportions of women of Christian faith reporting larger levels of awareness of the NHSBSP and screening process compared to women of Muslim faith. Due to elements of the Muslim faith guiding modesty, and the socio‐cultural stigma associated with women's health, barriers around breast health awareness are common as breast cancer is often an ‘unspeakable’ topic [4, 30, 31, 40, 77]. Stigma has been associated with cancer‐related health‐seeking behaviour by women, resulting in alternative medicine being used to delay diagnosis, reducing the chance of survival [41, 42]. Religious and cultural influences of cancer diagnoses may influence health beliefs and acceptance and, therefore, should be a consideration for health education and practice [29, 30, 31].

Whilst this study identified that women from South Lancashire were more likely to attend breast screening compared to East Lancashire, it is important to consider the disproportionate demographics represented in this study. Whilst in a previous study, British Pakistani women from East Lancashire have reported difficulties in understanding breast screening information and the ability to make informed decisions accessing healthcare [57], not all areas in East Lancashire have a similar demographic representation [78]. It is, therefore, important to consider the demographics, cultural influences and health education needs in a particular locality to better target screening awareness initiatives [79].

### 4.2 Barriers and Facilitators to Breast Screening

The results of this study suggest a need for targeted person‐centred health awareness. Previous studies have acknowledged various aspects of targeted awareness needs (e.g., language‐specific leaflets or a more accessible clinic) [52]. However, it is clear that challenges within each community vary widely, with work being needed to promote acceptance, remove taboos and open up dialogue and acceptance of cancer‐related health screening [12, 30, 32, 42, 44, 48, 54, 77, 80]. Within ethnic communities where women's health has often been seen as something to be kept private, creating a safe environment for women to also discuss their own views and concerns related to breast screening and breast health is pivotal to promoting person‐centred health promotion [81].

Public policy was highlighted in the present study as an area for improvement. Whilst the NHSBSP has a positive effect on breast screening awareness in age‐eligible women, there is still concern amongst some women that both younger women ( < 50) and older women ( > 70) should be included in this. Whilst there are challenges posed by breast density using current techniques in younger women, the results of this survey indicated that many older women were not always aware they were eligible to self‐refer for continued monitoring. Previous studies have also shown that some women believed they were not at risk for breast cancer because they were not eligible for breast screening [77]. Whilst the idea of increasing the age range for screening has also been supported by previous literature [55], this requires national policy change. Results from this study suggest it may be more pertinent to promote self‐referral to older women as part of local strategies for continued health education.

### 4.3 Limitations

The limitations of this study include the limited demographic represented not being fully aligned to the exploratory population sample. To allow further subgroup analysis on socio‐cultural influences and across protected characteristics (including disability/learning difficulties and language) on screening, future studies should consider specific strategies to encourage response rates and representation from all areas of the community. Whilst communities are often unjustly labelled ‘hard to reach’, if future studies encouraged co‐creation with the community to increase representation and equip communities to be able to better engage with health awareness initiatives, there would be a greater likelihood of success.

The present study established potential bias in the use of GPPAQ with older/retired or unemployed women. Whilst mobility issues due to ageing could explain the decreased ability to perform physical exercise, which leads to less physical activity performed [82], it is important that an appropriate tool is used to capture the true essence of physical activity amongst women to avoid bias. As physical activity has been shown to be one of the major risk factors in causing breast cancer, awareness of current activity levels will also potentially help to better target awareness interventions.

Future studies should ensure demographic representation to truly investigate the association between ethnic subgroups and breast knowledge, using a larger sample size for statistical analysis. Interventions and strategies to remove barriers and improve knowledge should also be developed from individual aspects up to public policy. With these areas targeted, breast health for women in Lancashire could be improved.

### 4.4 Study Implications

It is important to note that this study acknowledges the potential bias in reliability or accuracy offered by the GPPAQ, primarily due to the conscious bias implied by assuming retired or unemployed individuals are inactive. Therefore, future studies should avoid using this tool. Geographical area, faith and ethnicity significantly influenced the likelihood of women attending screening (MSES), highlighting the need for targeted interventions.

The socioecological model has had some use internationally when exploring barriers to mammography [83]. However, this study is the first to adopt a socioecological framework to highlight barriers to accessing breast screening in the United Kingdom, highlighting the need to address barriers at more than just the individual or organisational level. Fear of breast cancer did not significantly vary according to protected characteristics. However, perceived breast screening awareness and understanding were influenced by personal characteristics. Therefore, the findings of this study suggest health organisations should promote a person‐centred health awareness approach which should be adopted to increase breast screening uptake. Implementing the socioecological model for breast screening awareness by removing taboos at an individual, interpersonal and community level may help to create an accessible, inclusive open dialogue about cancer‐related health screening in communities.

Future interventions and strategies may look to adopt a community co‐creation approach with representative groups to improve strategies for reducing regional health inequities driving screening uptake. Ensuring adequate demographic representation of specific protected characteristics with higher response rates may also help to further explore strategic factors to influence breast screening uptake.

## 5 Conclusion

There has been limited previous research examining breast screening behaviours in Lancashire. This study was the first to use a mixed‐methods questionnaire to understand the behaviour, awareness, barriers and facilitators to breast screening in Lancashire to help guide trust‐based health promotions and initiatives. Age, ethnicity, faith and geographical location have all been found to influence breast screening behaviour significantly, highlighting the need to consider these factors in future initiatives to address the below‐national‐average breast screening uptake rates in Lancashire [3, 5]. This study highlighted that increasing cultural competency and inclusive practice amongst organisations may help to eradicate some of the barriers experienced. Whilst there is often a perception that end‐users are not engaging with screening services, the use of the socioecological model has highlighted that the responsibility to reduce or eradicate barriers to breast screening is collective and multifaceted.

## Author Contributions

**Yik Nok Bryan Lee:** writing – original draft, formal analysis. **Alexander Montasem:** validation, methodology, investigation, funding acquisition, writing – review and editing. **Lauren Haworth:** conceptualization, funding acquisition, writing – original draft, methodology, validation, formal analysis, supervision, writing – review and editing, data curation. **Jonathan Sinclair:** formal analysis, writing – review and editing, writing – original draft, methodology. **Kim McGuire:** formal analysis, writing – review and editing, funding acquisition, conceptualization, supervision. **Ambreen Chohan:** conceptualization, investigation, funding acquisition, writing – original draft, writing – review and editing, visualization, validation, methodology, formal analysis, project administration, resources, supervision, data curation.

## Conflicts of Interest

The authors declare no conflicts of interest.

## Data Availability

The authors have nothing to report.
